# Inhibitory Effect of Orally Administered 5-Aminolevulinic Acid on Prostate Carcinogenesis in the FVB-Transgenic Adenocarcinoma of a Mouse Prostate (FVB-TRAMP) Model

**DOI:** 10.31557/APJCP.2020.21.12.3743

**Published:** 2020-12

**Authors:** Kenta Onishi, Makito Miyake, Yoshihiro Tatsumi, Shunta Hori, Yasushi Nakai, Sayuri Onishi, Yusuke Iemura, Takuya Owari, Yoshitaka Itami, Kota Iida, Satoshi Anai, Nobumichi Tanaka, Keiji Shimada, Kiyohide Fujimoto

**Affiliations:** 1 *Department of Urology, Nara Medical University, 840 Shijo-cho, Kashihara city, Nara 634-8521, Japan. *; 2 *Department of Prostate Brachytherapy, Nara Medical University, 840 Shijo-cho, Kashihara, Nara 634-8522, Japan. *; 3 *Department of Pathology, Nara City Hospital, 1-50-1 Higashi kidera-cho, Nara city, Nara 630-8305, Japan. *

**Keywords:** Prostate cancer, 5-aminolevulic acid, chemoprevention, apoptosis, FVB-TRAMP

## Abstract

**Background:**

5-aminolevulinic acid (5-ALA) is a constituent of mitochondrial electron carriers, heme and cytochrome c, which are crucial for aerobic energy metabolism and cell apoptosis. We investigated the chemopreventive efficacy of 5-ALA against prostate cancer using the FVB-transgenic adenocarcinoma of mouse prostate (FVB-TRAMP) model.

**Methods:**

Samples were collected from 24 FVB-TRAMP mice at 12 and 20 weeks of age (named the first and second sets, respectively). Sixteen mice (from the first set) were randomly allocated into 3 treatment groups: 1) control (no treatment), 2) low dose of 5-ALA (30 mg/kg/day), and 3) high dose of 5-ALA (300 mg/kg/day). Similarly, 8 mice were divided into 2 treatment groups: 1) control and 2) high dose of 5-ALA (300 mg/kg/day). 5-ALA was orally administered to mice before cancer onset, from 6 weeks of age.

**Results:**

In the control group, prostate cancer was pathologically detected in 33 and 50 % of mice at 12 and 20 weeks, respectively, while 25% of 12-week old mice in the low-dose group were affected and none of the high-dose group mice developed prostate cancer. Immunohistochemical analysis showed higher expression of cytochrome c oxidase subunit 4 (COX4) in the prostate gland of the high-dose group compared to the control (P = 0.018). Similarly, enzyme-linked immunosorbent assay using lysed prostate tissue revealed higher amounts of cytochrome c in the prostate of the high-dose group compared to the control (P = 0.021). Furthermore, western blot analysis showed higher level of cleaved caspase-3 in mice in the high-dose group diagnosed with high-grade prostatic intraepithelial neoplasia.

**Conclusion:**

Our results suggest that oral 5-ALA may support the functional expression of mitochondrial cytochrome c and COX4, leading to caspase 3-dependent apoptosis in carcinogenesis in FVB-TRAMP mice. Future clinical studies are warranted to confirm the chemopreventive value of 5-ALA in prostate carcinogenesis.

## Introduction

Prostate cancer is one of the most common malignant diseases in men and is a major cause of mortality. Almost 1.3 million new cases of prostate cancer and 359,000 prostate cancer-associated deaths worldwide have occurred in 2018 (Bray et al., 2018). Patients in the early stage of prostate cancer can opt for any treatment option, and many of them are cured. However, once a patient reaches the castration-resistant state of the disease, no curative treatment option is available. Recently, much attention has been paid to agents useful in preventive strategies against prostate carcinogenesis. The transgenic adenocarcinoma of mouse prostate (TRAMP) model of prostate cancer was utilized to examine the chemopreventive effect of these agents since these tumors occur in the prostate epithelium and the histopathology of the tumor tissue closely mimics human prostate cancer.

Accumulating evidence suggests that the apoptosis response is closely linked to cancer prevention (Turrini et al., 2015). However, little information is available on the apoptotic effects of 5-aminolevulinic acid (5-ALA). This study focused on the potential of 5-ALA as a chemopreventive agent using the FVB-TRAMP model. 5-ALA is a naturally occurring amino acid found in plants and animals, and represents the first product of the porphyrin synthetic pathway with heme and cytochrome as the final products (Peng et al., 1997). 5-ALA plays an important role in the production of energy in the electron transport system through activation of mitochondria, enhancement of metabolism, and the anti-oxidant effect associated with the induction of radical scavengers such as heme oxygenase-1 (HO-1) (Miyake et al., 2014). In recent years, many studies have highlighted the usefulness of 5-ALA as an adjuvant in cancer treatment, such as in the capacity of medication that prevents side effects associated with chemotherapy (Terada et al., 2013) and resistance to anti-cancer drugs (Saito et al., 2013), and for radiosensitization (Yamamoto et al., 2015). In this context, we have previously reported that 5-ALA radiosensitizes prostate cancer tumor tissues and confers radioprotection to normal pelvic organs against radiation therapy (Miyake et al., 2019). However, few studies have reported the effect of carcinogenic suppression or anti-cancer effect of 5-ALA. In this study, we investigated whether orally administered 5-ALA has inhibitory effects on the carcinogenesis and progression of prostate cancer in an FVB-TRAMP model. 

## Materials and Methods

*FVB-TRAMP model*

Animal care was conducted in compliance with the recommendations of the Guide for Care and Use of Laboratory Animals (National Research Council). This study was approved by the Animal Facility Committee (protocol ID: 12126) and the Committee for Recombinant DNA Experiments (protocol ID: 177) at Nara Medical University. Experiments were carried out using an orthotopic carcinogenesis mouse model, namely the transgenic adenocarcinoma of mouse prostate (TRAMP) model in a FVB background. TRAMP heterozygous males were generated by mating TRAMP+/− females (C57BL/6 background) with non-transgenic FVB males. In TRAMP mice, the minimal rat probasin (rPB) regulatory sequence targets the SV40 early gene (T and t; Tag) expression speciﬁcally to the prostatic epithelium (Greenberg et al., 1995; Chiaverotti et al., 2008). The rPB-SV40 T transgene was identified using DNA extracted from tail samples. Polymerase chain reaction (PCR) was used to detect a 650-bp product using the following set of primers: 5’-GCGCTGCTGACTTTCTAAACATAAG-3’ 

(Pb-1, forward) and 

**Figure 1:**
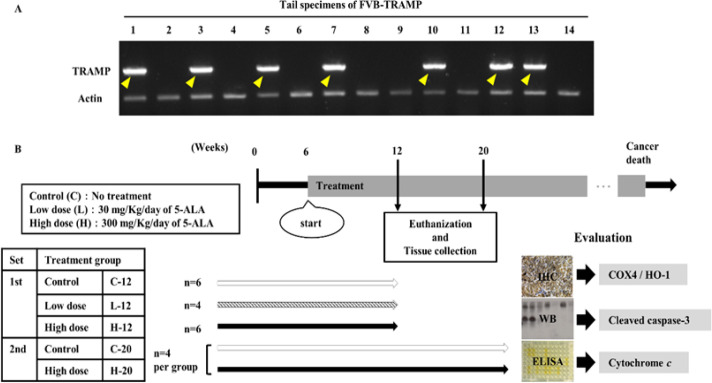
(A) Representative image from PCR for validation of the FVB-TRAMP model. Mouse IDs (1–14) are indicated at the top. DNA templates were amplified using primers (650 bp amplicon) targeting TRAMP. The primer was defined as positive for TRAMP and mice no. 1, 3, 5, 7, 10, 12, and 13 (highlighted by yellow arrowheads) were found to be TRAMP-positive. (B) Schematic diagram illustrating the study workflow. Mice were treated with orally administered 5-ALA or PBS from 6 weeks of age. The drug (or vehicle) was provided for 6 or 14 weeks until 12 or 20 weeks of age

5’-GAGCTCACGTTAAGTTTTGATGTGT-3’ (SV40Tag, reverse). The thermocycler was run for 35 cycles at 94°C for 3 min, 94°C for 30 s, 62°C for 1 min, 72°C for 3 min, and maintained at 4°C afterward (Shimada et al., 2013). Figure 1A shows a representative image from PCR that confirms the identity of the FVB-TRAMP mice.

*Study design*

Two sets of experiments with a total of 24 mice were performed (Figure 1B). In the first set, mice received oral administration of vehicle (phosphate-buffered saline) as a control (C-12 group, n = 6), low dose (estimated 30 mg/kg/day) of 5-ALA (L-12 group: n = 4), or high dose (estimated 300 mg/kg/day) of 5-ALA (H-12 group: n = 6) from 6 to 12 weeks of age (for 6 weeks). In the second set, mice received oral administration of vehicle as a control (C-20 group, n = 4) or a high dose (estimated 300 mg/kg/day) of 5-ALA (H-20 group: n = 4) from 6 to 20 weeks of age (for 14 weeks). 5-ALA hydrochloride was purchased from Sigma-Aldrich (St. Louis, MO, USA). All mice were euthanized by cervical dislocation at the end of the treatment period. Major organs (prostate, bladder, seminal vesicles, lungs, and liver) were dissected and subjected to macroscopic examination for viable abnormalities. Genitourinary (GU) organs including prostate, bladder, and seminal vesicles were weighed. The dietary intake and body weight of mice were monitored daily and the experiment was terminated in case of a marked loss in appetite and weight. If weight loss ≤ 20 or 25 % occurred within a period of 2 to 3 or 7 days, respectively, during the treatment period, euthanasia was performed.

*Hematoxylin and eosin (H&E) staining*

For pathological examination of prostate tissues, H&E staining was performed according to standard laboratory protocols. The tissues were fixed with 10% neutral buffered formalin and embedded in paraffin. The paraffin block was then sliced into 3-μm slices. For pathological examinations, H&E-stained slides were reviewed by a pathologist (K. Shimada) with expertise in prostate cancer diagnosis.

*Immunohistochemical (IHC) staining*

IHC staining was performed to evaluate the expression levels of cytochrome c oxidase subunit 4 (COX4) and HO-1 in prostate specimens from the first set of mice (C-12, L-12, and H-12 groups). Tissue sections were obtained from paraffin-embedded tissue blocks, which were sliced and placed on Superfrost Plus microscope slides (Thermo Fisher Scientific, Yokohama, Japan). The tissue sections were deparaffinized, and antigen retrieval was carried out in citric acid buffer (pH 6.0) in an autoclave. We performed IHC staining using a Histofine ABC kit (Nichirei, Tokyo, Japan). The slides were treated with 1% hydrogen peroxide in methanol to block endogenous peroxidase activity and incubated overnight at 4°C with COX4 (Abcam, Cambridge, UK; rabbit monoclonal, dilution 1:100) and HO-1 (BD biosciences, San Jose, CA, USA; mouse monoclonal, dilution 1:100) antibodies. The slides were then counterstained with hematoxylin, dehydrated, and mounted on a cover slide. Three randomly selected high-power fields (x400 magnification) were utilized for evaluating the percentage of COX4- or HO-1-positive cells in normal or cancerous prostate epithelium cells and the obtained values were averaged in each case.

*Enzyme-linked immunosorbent assay (ELISA)*

The level of cytochrome c in tissues of the second set (C-20 and H-20 groups) of mice were estimated. For this, tissue lysates obtained by homogenizing prostate samples were used in a Quantikine ELISA Rat Cytochrome c Immunoassay (R&D Systems, Minneapolis, MN, USA) according to the manufacturer’s protocol.

*Western blot (WB) analysis*

Total cellular protein lysates were prepared as described previously (Hori et al., 2018). Protein was extracted from prostate tissues diagnosed with high-grade prostatic intraepithelial neoplasia (PIN) in each group of the second set (C-20 and H-20 groups) and 10 μg of total protein was acquired. In brief, samples were homogenized and incubated in a lysis buffer (250 mmol/L Tris-HCl (pH 6.8), 2% sodium dodecyl sulfate (SDS), and 10% glycerol) and proteinase inhibitor cocktail (Sigma-Aldrich, St. Louis, MO, USA) for protein extraction. The membranes were incubated for 1 h with anti-cleaved caspase-3 rabbit monoclonal antibody (Cell Signaling Technology Inc., Danvers, MA, USA; dilution 1:1000) and anti-actin mouse monoclonal antibody (dilution 1:10,000) as an internal loading control, followed by a 1-h incubation with horseradish peroxidase-conjugated anti-rabbit IgG (1:5,000) or anti-mouse IgG antibody (1:20,000) (Santa Cruz Biotechnology, Dallas, TX, USA).

*Statistical analysis*

All statistical analyses were performed and figures were generated using the Prism software 5.00 (GraphPad software, San Diego, CA, USA). Cochran-Armitage tests were conducted for trend analysis of pathological diagnosis among groups. Comparisons between 2 and 3 treatment groups were performed using MannWhitney U test and KruskalWallis test, respectively, followed by a Scheffe test. A P value <0.05 was considered statistically significant.

## Results

*Inhibitory effect of orally administered 5-ALA in prostate carcinogenesis*

**Figure 2:**
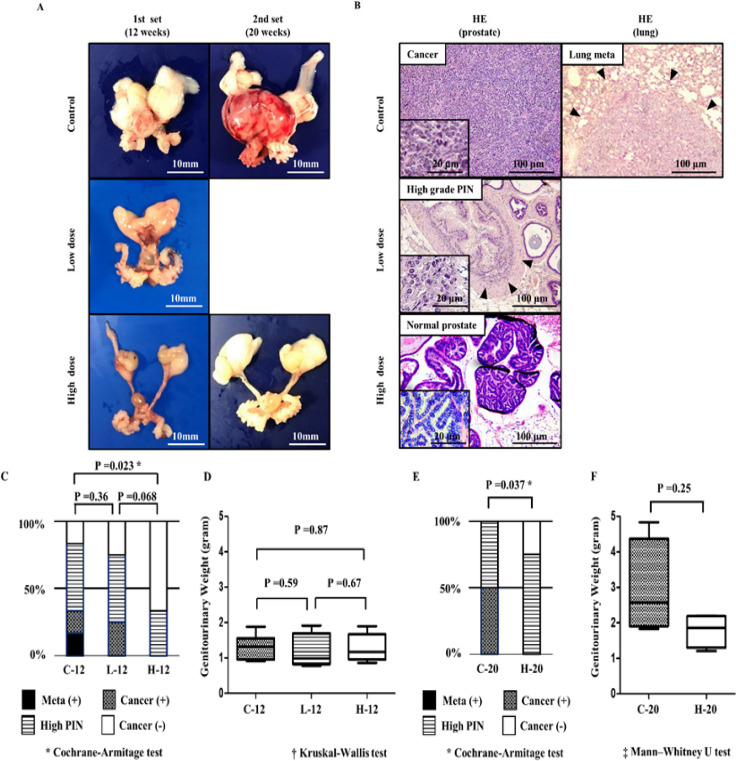
(A) Photographs of representative genitourinary (GU) samples of each group. (B) Representative images of Hematoxylin and Eosin (H&E) staining of tissues obtained from each group of mice. Histological section of the control group displays poorly differentiated tumor with noticeable cellular atypia and metastasis to the lung (arrowheads). Histological section of the low-dose group displays high grade PIN (prostatic intraepithelial neoplasia) with papillary proliferation of glandular epithelium (arrowheads) and low cellular atypia. Histological section of the high-dose group displays normal prostate epithelium without cellular atypia. (C) Histological evaluation of the incidence of cancer, high grade PIN, and normal prostate tissue in the first set of mice. Cochrane-Armitage test was used to evaluate differences in tumor grade level between control and 5-ALA-administered groups. There were significant differences between the C-12 and H-12 groups (P = 0.023). (D) GU weights of each group in the first set. No significant difference was observed among the 3 (C-12, L-12, and H-12) groups. (E) Histological evaluation of the incidence of cancer, high grade PIN, and normal prostate tissue in the second set. There were significant differences between the C-20 and H-20 groups (P = 0.037). (F) GU weights of each group in the second set. No significant difference was observed between the C-20 and H-20 groups

There was no significant treatment-induced difference in body weight of mice among the groups in both sets (data not shown). None of the mice showed weight loss due to disease progression or any obvious adverse (toxic) events caused by the oral administration of 5-ALA. Macroscopically, some prostate samples in the C-20 group were observed to be large due to tumor progression (Figure 2A). Figure 2B shows representative images of H&E-stained tissue sections from each group. In the first set, pathological examination revealed that 2 (33.3%) out of 6 mice in the C-12 group had prostate cancer, 3 (50%) had high-grade PIN, and the remaining (16.7%) did not develop cancer. Moreover, lung metastasis from prostate cancer was detected in one mouse (16.7%). However, in the L-12 group (n=4), one (25%) had prostate cancer and 2 had high-grade PIN; and in the H-12 group (n=6), 2 had high-grade PIN and the remaining four (66.7%) did not have prostate cancer. The Cochrane-Armitage test revealed a significant difference between the C-12 and H-12 groups (P = 0.023; Figure 2C).The median GU weight and range of the C-12, L-12, and H-12 groups were 1.32 (range, 0.91-1.45), 0.99 (range, 0.88-1.92), and 1.17 (range, 0.86-1.59) g, respectively (Figure 2D). No significant difference in GU weight was noted among the different groups. In the second set, 2 (50%) of 4 mice in the C-20 group had prostate cancer and 2 (50%) had high-grade PIN. On the other hand, 3 out of 4 mice had high-grade PIN and no mouse had evidence of prostate cancer in the H-20 group. There was a significant difference in prostate cancer progression between the C-20 and H-20 groups (P = 0.037; Figure 2E). The median GU weight of the C-20 and H-20 groups was 2.57 (range, 1.83-4.84) and 1.87 (range, 1.21-2.2) g, respectively. No significant difference in GU weight was observed between the two groups (P = 0.25; Figure 2F).

*Increased mitochondrial activity in prostate tissues on 5-ALA administration*

**Figure 3:**
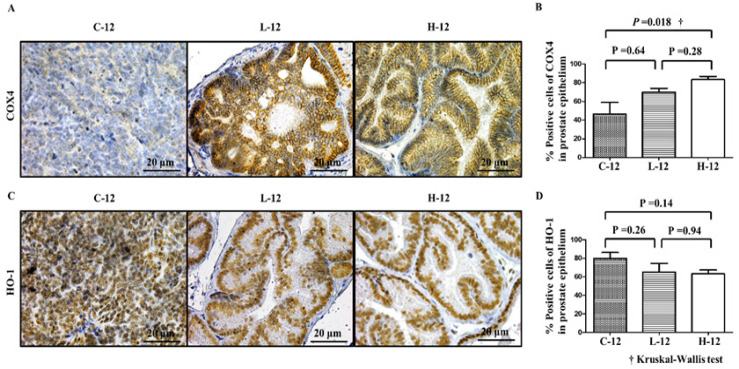
(A) Representative images of immunohistochemical (IHC) staining for cytochrome c oxidase subunit 4 (COX4). (B) COX4-positive cells were counted and represented as a percentage. The expression level of COX4 in the prostate tissues of the L-12 group was not significantly difference compared to that in the C-12 group (P = 0.64), although that in the H-12 group was significant higher than that in the C-12 group (P = 0.018). (C) Representative images of immunohistochemical (IHC) staining for heme oxygenase-1 (HO-1) in prostate tissues. (D) There was no significant difference in HO-1 expression among the 3 (C-12, L-12, and H-12) groups

The expression of COX4 is known to represent cellular mitochondrial activity (Ogura et al., 2011). Figure 3A shows representative IHC images of COX4 expression in each group. There was no significant difference in the COX4 expression level in prostate epithelial cells between the C-12 and L-12 groups, while a significantly higher level of COX4 was observed in the H-12 groups compared to both C-12 and L-12 groups (Figure 3B). 

HO-1 expression is believed to represent a marker for the anti-oxidative stress in cells resulting from the activation of mitochondrial function (Lawal et al., 2015). Figure 3C shows representative IHC images of HO-1 expression in each group. There was no significant difference in the expression of HO-1 among the 3 (C-12, L-12, and H-12) groups (Figure 3D).

*Apoptotic induction via the caspase-3 pathway by 5-ALA oral administration*

**Figure 4:**
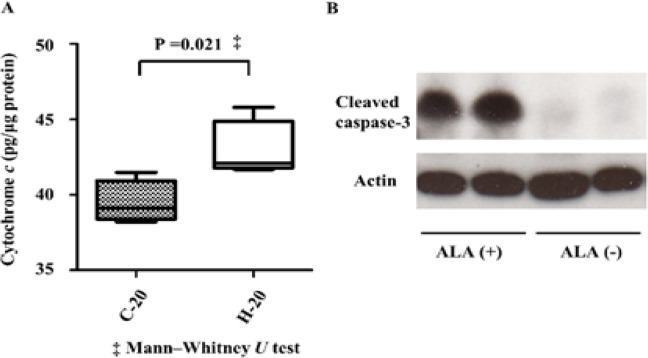
(A) Concentrations of cytochrome c in prostate tissues estimated by enzyme-linked immunosorbent assay (ELISA). The concentration of cytochrome c in the H-20 group was significantly higher than that in the C-20 group (P = 0.021). (B) Protein expression of cleaved caspase-3 in the TRAMP model diagnosed with high grade PIN by western blot analysis. The expression level of cleaved caspase-3 was higher in the prostate samples of the H-20 group compared to that in the C-20 group

We evaluated the amount of cytochrome c in prostate tissues collected from mice in the C-20 and H-20 groups (from the second set). An ELISA-based experiment using the lysate of the prostate tissues showed that the median cytochrome c level in tissues from the C-20 and H-20 groups was 39.1 (range, 38.2-41.5) and 42.1 (range, 41.7-45.8) pg/μg protein, respectively. A significantly higher level of cytochrome c was noted in the H-20 group compared to the C-20 group (P = 0.021; Figure 4A). Furthermore, the possible involvement of cleaved caspase-3 in apoptosis of the prostate epithelia in mice orally administered with 5-ALA was explored. For this, prostate tissues obtained from mice in the second set (C-20 and H-20 groups) with high-grade PIN were subjected to western blot analysis, which revealed the high expression levels of cleaved caspase-3 in prostate samples of the H-20 group (Figure 4B). 

## Discussion

Efforts to reduce the incidence and delay the progression of prostate cancer assumes critical importance in the backdrop of the high incidence and mortality rates associated with prostate cancer worldwide. The preventive approach is ideal for prostate cancer, since the incidence of this form of cancer generally increases with age over 50 years. Many studies have examined the chemopreventive effect of drugs as a viable therapeutic strategy against prostate cancer using the TRAMP model. Various chemopreventive agents have been identified using the TRAMP model, including natural compounds such as green tea (Sartor et al., 2004; Adhami et al., 2004), genistein (Mentor-Marcel et al., 2001; Wang et al., 2007), fish oil (Saw et al., 2011), withaferin A (Suman et al., 2016), and broccoli sprouts (Beaver et al., 2017). However, no study has reported the anticancer properties of 5-ALA in the TRAMP model. The present study was designed to explore whether oral administration of 5-ALA could inhibit or delay the development of prostate cancer in the FVB-TRAMP model.

5-ALA is an amino acid naturally present in every living cell of the human body. 5-ALA is produced in the mitochondria as the first product of the porphyrin synthesis pathway, represents a precursor for heme biosynthesis, and helps in upregulating mitochondrial functions. As 5-ALA is a natural substance in food and beverages such as wine and vinegar, it has low toxicity and no side effects of 5-ALA have been reported in normal cells (Ishizuka et al., 2011). In addition, no apparent toxicity was observed in mice administered with 5-ALA in the present study. There have been few reports on the effect of carcinogenic suppression or anti-cancer effect of 5-ALA itself. Sugiyama et al. (2014) reported that administration of 5-ALA inhibits the Warburg effect, protecting cancer cells against oxidative stress-mediated apoptosis and inducing cancer cell death in vitro. Our results demonstrated that oral administration of 5-ALA from 6 weeks of age in the FVB-TRAMP model significantly reduced tumor burden, relative to that in the control group. We hypothesize that the mechanism of cancer inhibitory effect exhibited by 5-ALA involves the induction of apoptosis in the prostate tissues. This study showed that 5-ALA activates mitochondrial function in the prostate, since IHC staining showed high expression of COX4 in the prostate tissues of the 5-ALA-administered mice. COX4 is a mitochondrial transmembrane protein complex and its activity in the electron transfer chain is fundamentally important in aerobic energy metabolism. This enzyme catalyzes electron transfer from cytochrome c to molecular oxygen, reducing the latter to water, and yields substantial energy that drives the formation of a proton gradient that is then employed to synthesize ATP (Capaldi, 1990). Ogura et al. (2011) reported that oral administration of 5-ALA increases COX4 activity in the mouse liver, and demonstrated higher ATP levels in 5-ALA-treated mouse livers compared to those in PBS-administered controls (Ogura et al., 2011). In the present study, COX4 activity could be presumed to have increased in the mouse prostate after 5-ALA administration. Activated mitochondria in the prostate could enhance mitochondrial membrane permeability and release cytochrome c into the cytoplasm. The released cytochrome c would then activate caspase-3 and induce apoptosis. Overall, activated mitochondria in prostate tissues could promote apoptosis on oral administration of 5-ALA. The present study demonstrated the carcinogenesis inhibitory effect of 5-ALA, which might induce apoptosis in prostate tissues by restoring normal metabolic activities in the FVB-TRAMP model. 

This study has some limitations. First, the sample size of the FVB-TRAMP model was small. It is necessary to conduct an analysis with a larger cohort of mice, as there are individual differences among the FVB-TRAMP model in terms of the time period of carcinogenesis and the speed of cancer development. Second, there was a problem with the method of administering 5-ALA to mice, which was provided freely to mice via a lightproof water bottle in this study. Therefore, the dosage for each mouse could not be exactly measured. Oral gavage should also be considered. Third, we evaluated the mechanism of apoptosis by only quantifying cytochrome c level. Since the apoptotic process is known to be associated with reduction in mitochondrial membrane potential, further studies are needed on changes in both the cytochrome c level and mitochondrial membrane potential to clarify the chemopreventive effect of 5-ALA. Fourth, the function of HO-1 could not be elucidated. HO-1 participates in maintaining cellular homeostasis and plays an important protective role in tissues. On the other hand, HO-1 activation plays a role in carcinogenesis and can potentially influence the growth and metastasis of tumors (Jozkowicz et al., 2007). The activation of mitochondrial function by 5-ALA administration was initially hypothesized to have resulted in the production of a large amount of heme and upregulation of HO-1, an antioxidant stress gene, which could suppress inflammatory pathways to decompose excess heme. However, there was no difference in the expression of HO-1 with or without 5-ALA administration. Further experiments are therefore needed to clarify the relationship between HO-1 and carcinogenesis.
